# Stricture of the Urethra—Treatment of and Operation for

**Published:** 1860-07

**Authors:** S. Wilkins

**Affiliations:** Vandalia, Ill.


ARTICLE 3.

### STRICTURE OF THE URETHRA. TREATMENT OF AND OPERATION FOR.

BY S. WILKINS, M. D., VANDALIA, ILL.

January 4th, 1859, I commenced treating the following case, the subject of which I find to be thirty-seven years old, weight 165 pounds, (when in health,) and highth five feet, ten inches; temperament, nervous-sanguine; occupation, a farmer.

*History.*—May 1858, (as I learn,) he received a kick from a man which wounded the urethra. From the physicians who were called to treat the case, one of them when the accident first occurred, and the other soon after that time, I had learned that effusion in the scrotum and perineum took place, which was followed by suppuration. His condition when I saw him, was as follows:

Pale, emaciated, constipated; urine of a reddish color and appetite usually bad; sharp and anxious countenance; tongue coated with a dirty yellow; rigors and night sweats; passes no urine through the penis, but on examination a stricture is found about five or five and a half inches from the extremity of that organ in the urethra. Behind this stricture was a urinary fistula through which the urine all passed. Around this fistula the scrotum was drawn up and appeared adherent to the urethra. Being unable to control his urine, it dribbled away with irregularity. It was caught and absorbed by cloths, and its odor made it very disagreeable to be in the room. His condition was pitiable indeed. He was willing to submit himself to any treatment or risk which would promise the least prospect of cure.

Appropriate constitutional treatment was instituted, and his general condition soon improved. The local treatment consisted in that which is usual in such cases, viz: the introduction of catheters, bogies, and sounds, down to the stricture through which it was impossible to pass, although I took impressions with a wax bogie, learned the direction which the channel appeared to take, and made repeated trials. These trials were made with gum catheters and bogies of various sizes, flexible and inflexible, all of which had been bent in various directions, to suit the case if possible. By the introduction of catheters and bogies occasionally, the urine, to some extent, was made to flow out of the urethra at the extremity. This, however, would not continue long at a time, when the urine, or most of it, would pass through the stricture. Cauterization of the stricture was resored to with the nitrate of silver. It was introduced, protected on the side, and held in contact with the stricture for one minute, when it was withdrawn. Considerable pain followed its introduction, which would become so severe soon after as to require sedatives in large doses, to allay it. I had learned, from a wax impression, that the stricture was more in the left than the right side, which was taken advantage of, both in the introduction of the catheters and bogies, and in the application of the caustic. Although a bogie or catheter would sometimes appear to enter and become grasped by the stricture, I was never able to introduce any instrument into the bladder. It appeared as though a part of the stricture might have been obliterated, as the instruments seemed to pass to a greater extent in the penis. Repeated stretching, however, might have elongated that organ. Not being able to decide this question, each one of the profession can decide it for himself. Having treated the case a sufficient length of time, and having consulted several surgeons in relation to it, I determined on the following operation, which was performed June 10th, 1859:

*Operation.*—Drs. Haller, Hickman, Bassett, and Tidball, being present, and kindly lending me their assistance, the patient was placed as in the operation for lithotomy. A metalic sound was introduced in the urethra down to the stricture, and supported there by an assistant. The scrotum was raised and supported, so as to be out of the way. Being under the influence of chloroform, an incision in the perineum was made, extending longitudinally over the urethra about two inches in length. This was gradually deepened in the direction of the sound till it was reached. It was found about one inch or over from the surface. In the region of vessels the back of the bistoury was used in place of the edge, and when cutting through the stricture, the point of the knife was directed to it by having the index finger of the left hand introduced into the wound, feeling for the point of the sound, and keeping the end of the finger as near to it as possible. The bistoury was directed to the stricture by the side of the finger, with its edge in the wound and back towards the scrotum, in order to lessen the chances of wounding the vessels. An instrument was introduced in the bladder, by finding the place from which the urine flowed; this latter having been retained for that purpose. The stricture was exceedingly hard to cut, so much so that the assistants could both hear and feel the cutting. When the stricture was opened and the sound passed through, it was withdrawn and a gum catheter of good size introduced in its place, when it was introduced on into the bladder, along a director which was held in the urethra, at the wound, for that purpose. The director was then withdrawn, the wound closed over the catheter, cold water dressing compress and bandage applied. The hemorrhage was considerable, but the most of it was venous, and it was not very hard to stanch. The catheter was retained in its place by narrow strips of adhesive plaster wound spirally rounnd the penis, and attached to the catheter. The urine soon passed quite freely through the catheter.

The operation was a lengthy one, but was well borne, and the shock inconsiderable. The day after the operation the wound was examined, and the catheter was well surrounded with a provisional urethra, and but little of the urine passing through the wound. During the first two or three days the catheter has been out as many times, and re-introduced without much difficulty. Compresses have been kept on the sides of the wound in such a way as to keep the wound in juxtaposition at the bottom, from which it appears to be slowly healing up, but there is yet a considerable portion of the outer part of the wound yet ununited. The granlations are rather flabby and unhealthy, but stimulation by a solution of Nitrate of Silver, &c., has been applied with a hope of getting it to do better. It is now the 24th of June, and on the whole the case has done as well as could be expected. It is possible the wound might have been healed by the first intention, but it was purposely left open and kept so by introducing into it a wedge-shaped piece of sponge, partly for that purpose, and to prevent secondary hemorrhage, and is this did not occur, it appeared effectual for that purpose.

The suppuration in the urethra occurred during the second day, was moderately plentiful at first, but soon appeared to diminish.

It is now the 25th of May, 1860. The case has been well for several months, and the cure is complete. The man is attending to active business every day.

